# From Synovial Tissue to Peripheral Blood: Myeloid Related Protein 8/14 Is a Sensitive Biomarker for Effective Treatment in Early Drug Development in Patients with Rheumatoid Arthritis

**DOI:** 10.1371/journal.pone.0106253

**Published:** 2014-08-28

**Authors:** Ivy Y. Choi, Danielle M. Gerlag, Dirk Holzinger, Johannes Roth, Paul P. Tak

**Affiliations:** 1 Division of Clinical Immunology and Rheumatology, Academic Medical Center/University of Amsterdam, Amsterdam, the Netherlands; 2 Department of Paediatric Rheumatology and Immunology, University Children’s Hospital Muenster, Muenster, Germany; 3 Institute of Immunology, University Hospital Muenster, Muenster, Germany; University Hospital Jena, Germany

## Abstract

**Objective:**

The change in number of CD68-positive sublining macrophages in serial synovial biopsies has been successfully used to discriminate on the group level between effective and ineffective treatment during early drug development in rheumatoid arthritis (RA) patients. Measurement of a soluble biomarker would clearly have practical advantages. Therefore, we investigated the sensitivity to change of myeloid related protein (MRP)8/14 in serum.

**Methods:**

139 RA patients who received known effective biologics (infliximab, adalimumab and rituximab) and 28 RA patients who received placebo/ineffective therapies were included. MRP8/14 levels were analyzed in baseline and follow-up serum samples and the standardized response mean (SRM) was calculated to determine the sensitivity to change of MRP8/14 in comparison to C-reactive protein (CRP) levels and the disease activity score evaluated in 28 joints (DAS28).

**Results:**

In patients treated with effective treatment, the SRM for MRP8/14 was moderate (0.56), but in patients treated with placebo/ineffective treatment the SRM was 0.06, suggesting that this biomarker is perhaps not susceptible to placebo effects in proof-of-concept studies of relatively short duration. In contrast, the SRM for DAS28 was high for effective treatment (1.07), but also moderate for ineffective treatment (0.58), representing the placebo effect. The SRM for CRP was low in the effective (0.33) and ineffective (0.23) treatment groups.

**Conclusion:**

These data support the notion that quantification of changes in MRP8/14 serum levels could be used to predict potential efficacy of novel antirheumatic drugs in an early stage of drug development. A positive result would support the rationale for larger, conventional clinical trials to determine whether the effects are clinically relevant.

## Introduction

Rheumatoid arthritis (RA) is a chronic systematic inflammatory disease affecting the synovial tissue in multiple joints. In spite of significant improvement in treatment, there is still a significant subset of patients that does not respond sufficiently to currently available medicines. Thus, there is still a need for treatments with a novel mechanism of action, and several of these are currently in development. The conventional approach towards clinical trials during early drug development is however not sustainable due to the high costs involved in testing large numbers of patients to provide proof of mechanism; slow recruitment rates due to improved standard of care as well as the large number of competing clinical trials; and ethical considerations: why would one unnecessarily expose large numbers of patients to placebo or experimental drugs that are likely to be ineffective in light of the high attrition rates during early drug development?

Therefore, we have previously proposed a fundamentally different approach towards phase Ib/IIa clinical trials: small, high density-of-data clinical trials evaluating biomarkers associated with common final pathogenetic pathways known to be relevant for the disease, biomarkers associated with the proposed specific mechanism of action, and trends towards clinical improvement. [1] We have previously identified and validated the expression of CD68+cells in the synovial tissue of RA patients as a biomarker that is related to changes in clinical signs and symptoms independent of the primary mechanism of action. [2]–[4] We have recommended a rethinking of the decision to move ahead to large clinical trials when there is no trend towards clinical improvement, no specific effect related to the mechanism of action, and no change in CD68+ macrophage numbers in the synovium after treatment. [1] If this is the case, the drug might not hit the target effectively or the target may not be the right one. However, when there is a signal in at least one of these three variables, the next step would be to test the drug in conventional studies to determine whether the biological effect translates into clinically meaningful improvement. This screening approach, where the change in the number of CD68-positive sublining macrophages in serial arthroscopic synovial biopsy specimens is used to discriminate on the group level between effective treatment and ineffective during early drug development, has been successfully used to test a wide variety of experimental medicines, [1], [5] allowing early go/no go decisions related to further clinical development. Such proof of mechanism studies may not only help to screen for potential efficacy, but may also help to optimize the range of dosages to be tested in the phase II/III trials.

While synovial biopsy is a safe and generally well-tolerated procedure in experienced hands [6], a limitation of this approach is that it is only used in a limited number of centers. Moreover, serial synovial biopsy is mainly restricted to the knee, ankle and wrist joints, affecting recruitment as not all patients with active RA have clinical involvement of these joints. Thus, there is a clear need for a biomarker reflecting the changes in monocyte/macrophage infiltration and activation in the synovial compartment in response to treatment, but which can be measured in the peripheral blood to screen for potential efficacy on the group level during early drug development.

Myeloid-related protein (MRP)-8 and MRP-14 are calcium-modulated proteins that regulate myeloid cell function and control inflammation. The heterodimer MRP8/14 is released during the interaction of monocytes with activated endothelium, probably at sites of local inflammation. [7] Therefore, we investigated the sensitivity to change of this soluble biomarker in RA patients in small clinical trials of short duration, similar to how this would be used in early phase Ib/IIa proof of mechanism clinical trials.

## Materials and Methods

We investigated the changes after treatment with three known effective biologics: infliximab, adalimumab and rituximab; these open label prospective clinical trials have been described before. [8] Briefly, in the first cohort, 34 patients were treated with infliximab 3 mg/kg intravenously at baseline, week 2, week 6 and subsequently every 8 weeks. [9] In the second cohort (ISRCTN68762628), 85 patients received adalimumab 40 mg subcutaneously at baseline and every other week, [10] and in the third cohort (ISRCTN05568900), 20 patients were treated with intravenous infusions of 1000 mg rituximab at day 1 and 15 after premedication with 2 mg clemastine fumarate and 1000 mg acetaminophen but without methylprednisolone [11].

We also analyzed data obtained in 3 randomized, controlled clinical trials of treatment strategies that were shown to be ineffective in RA: C5a receptor-antagonist (C5aR; n = 6), [12] a human C-C chemokine receptor type 2 blocking antibody (CCR2; n = 8), [13] C-C chemokine receptor type 5 inhibitor (CCR5, n = 13), [5] and one patient treated with placebo in a randomized, double-blind, placebo-controlled study with abatacept (Clinicaltrials.gov NCT00420199) in patients with RA. [14] Targeting C5aR, CCR2 and CCR5 has been shown ineffective in RA in these placebo-controlled, phase Ib/IIa trials. [5], [12], [13] The studies were performed in the Academic Medical Centre/University of Amsterdam and approved by the Medical Ethics Review Board of the Faculty of Medicine (AMC-UvA). All patients gave written informed consent.

We collected serum samples at baseline and at a follow up visit after initiation of effective treatment or placebo/ineffective treatment. The follow up samples were the samples taken after baseline. For trials with effective treatment as well as C5aR antagonists and anti-CCR5 antibodies, the follow up samples were obtained 4 weeks after baseline. For the trial with anti-CCR2 antibodies, the follow up sample was taken at day 43. In the trial with placebo versus abatacept, the follow up sample was taken at day 113. Serum levels of MRP8/14 complexes were determined by double-sandwich enzyme-linked immunosorbent assay (ELISA) system, performed in the laboratory of the University of Münster, as described previously. [8], [15] For comparison with earlier studies, internal control sera were used as a reference in all ELISA studies. The readers of the laboratory assay were blinded for the clinical details and samples were analyzed in a random order. In addition, we measured serum C-reactive protein (CRP) serum levels at these time points and determined the disease activity score evaluated in 28 joints (DAS28-CRP).

### Statistical analysis

Continuous data were described as median and interquartile range (IQR) if not normally distributed. The chi-square test was used to compare categorical characteristics. Mann-Whitney U test was used to compare continuous variables. All statistical analyses were performed with SPSS 20.0 software (IBM Corp., Armonk, NY). A p-value <0.05 was considered statistically significant. The standardized response mean (SRM), a measure of sensitivity to change, was calculated by dividing the mean change over time by the standard deviation of that change. The SRM of the clinical scores (CRP and DAS28-CRP) and MRP8/14 were calculated to evaluate the ability of these measurements to detect changes over time in the effective treatment groups (infliximab, adaliumab and rituximab treatment) and in the ineffective/placebo group (patients who received placebo or treatment with C5aR antagonist, anti- CCR2 or anti-CCR5 antibodies). An SRM>0.8 is considered high, representing a change of at least four fifths of a standard deviation of the change in the score. An SRM of 0.5 is defined as having moderate potential to detect changes, and an SRM of 0.2 as having low potential.

## Results

One hundred thirty-nine patients treated with a known effective treatment and 28 patients treated with a placebo or ineffective treatment were included and analyzed in this study. Baseline characteristics of the patients treated with effective treatment or placebo/ineffective treatment are shown in Table 1. Age and gender were representative of typical RA cohorts. The response rate, as determined by the EULAR response, is as expected significantly higher in the group of patients treated with known effective treatment (p<0.0001). Tender and swollen joint counts of 68 joints (TJC68 and SJC68) at baseline were comparable between these groups. There were also no significant differences in baseline levels of erythrocyte sedimentation rate (ESR) and DAS28-CRP between the groups. Baseline CRP serum levels were significantly higher in the placebo/ineffective treatment group compared to the effective treatment groups (p = 0.029).

**Table 1:** Baseline characteristics of patients enrolled in the effective and placebo/ineffective treatment groups.

	Effective treatment (n = 139)	Placebo/ineffective treatment (n = 28)	p-value
Female, n (%)	106 (76.3)	18 (64.3)	0.186
Age (years)	55 (47–63)	51 (36–59)	0.097
EULAR responder, n (%)	110 (79.1)	10 (35.7)	<0.0001
TJC68	10 (6–17)	10 (4–12)	0.093
SJC68	7 (4–12)	8 (6–11)	0.633
ESR	22 (12–37)	28 (18–56)	0.122
CRP	9 (3.5–22)	13 (6–47)	0.029
DAS28-CRP	4.72 (4.05–5.87)	4.77 (4.41–5.46)	0.662

Median and interquartile range or percentages are shown. EULAR responder = good or moderate responder according to the European League Against Rheumatism Response, TJC68 =  tender joint count of 68 joints, SJC68 =  swollen joint count of 68 joints, ESR = erythrocyte sedimentation rate, CRP = C-reactive protein, DAS28-CRP = disease activity score based on CRP. P-value <0.05 was considered statistically significant. Significance of the comparison is determined by the chi-square test or the Mann-Whitney test.

### Sensitivity to change of MRP8/14 serum levels compared to DAS28 and CRP serum levels

**Figure 1:**
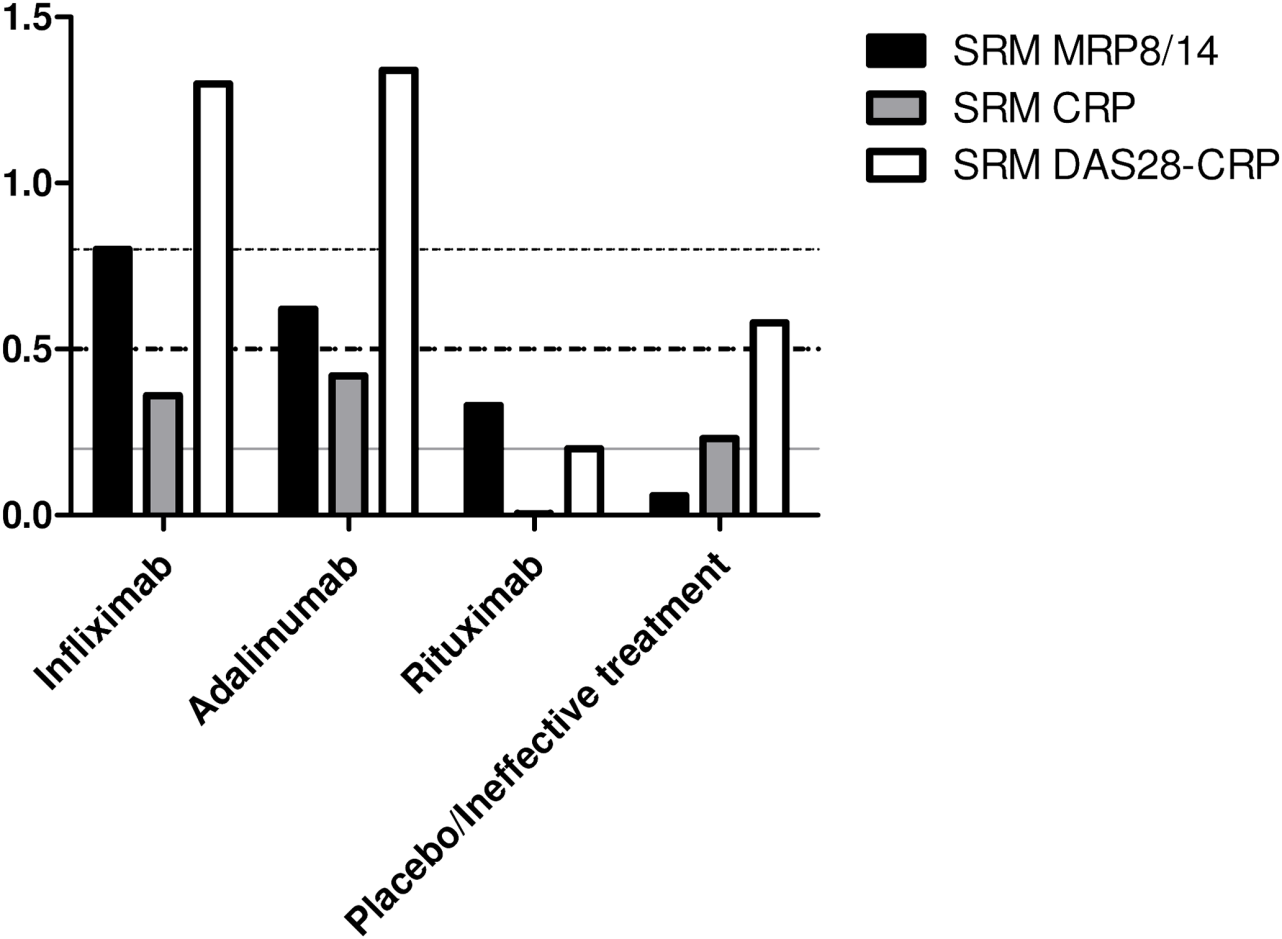
Sensitivity to change of MRP8/14 serum levels compared to DAS28 and CRP serum level. Standardized response mean (SRM) of the change in myeloid related protein 8/14 (MRP8/14), C-reactive protein (CRP) and disease activity score evaluated in 28 joints (DAS28-CRP) 4 weeks after treatment with infliximab (n = 34), adalimumab (n = 85), rituximab (n = 20) and treatment with placebo or ineffective therapy (n = 28). The solid line indicates the 0.2 SRM cut off point (low), the thick dotted line indicates the 0.5 SRM cut off point (moderate) and the thin dotted line indicates the 0.8 SRM cut off point (high).

In the first cohort (infliximab treatment), the SRM for MRP8/14 was 0.80 and the SRM for DAS28-CRP was 1.42 (Figure 1). Similarly, in the second cohort (adalimumab treatment), the SRM for MRP8/14 was 0.62 and the SRM for DAS28-CRP was 1.34 (Figure 1). The MRP8/14 biomarker exhibited consistently higher sensitivity to change than CRP in these anti-TNF cohorts, which was 0.36 and 0.42, respectively (Figure 1). In the third cohort (rituximab treatment), the SRM for MRP8/14 was 0.33 and the SRM for DAS28-CRP was 0.18 (Figure 1). In this cohort, the SRM for CRP was 0.00 (Figure 1). The SRM for MRP8/14 for the pooled effective treatment groups was 0.56, the SRM for DAS28-CRP was 1.07 and the SRM for CRP was 0.33.

In the studies with placebo/ineffective treatment, the SRM for MRP8/14 was 0.06, while the SRM for DAS28-CRP was 0.58 (Figure 1). The SRM for CRP in this group was 0.23 (Figure 1).

### Sensitivity to change of CRP remains low if only patients with higher CRP levels at baseline are included in the analysis

To test whether CRP may have higher sensitivity to change if only patients with higher CRP levels at baseline are included, we calculated the SRM for CRP if CRP at baseline >5 mg/L. The SRM for CRP in the infliximab, adalimumab and rituximab was 0.46, 0.54 and 0.00, respectively. The SRM for CRP for the pooled effective treatment groups remained low (SRM of 0.40) and also remained low for the ineffective/placebo group (SRM of 0.26).

### Sensitivity to change of MRP8/14 remains low in responders after ineffective/placebo treatment

As described in table 1, there was a 35.7% clinical response rate in the ineffective/placebo treatment group. When we calculated the SRM for MRP8/14 in EULAR good/moderate responders after known ineffective or placebo treatment, the sensitivity to change for MRP8/14 remained low (SRM of 0.12). In this group, the SRM for DAS28-CRP was 1.62 and the SRM for CRP was 0.35.

## Discussion

We investigated the potential of soluble MRP8/14 as a biomarker to screen for initial evidence of efficacy on the group level during early drug development in small proof-of-mechanism trials in RA patients. The SRM for MRP8/14 in the two anti-TNF cohorts indicates a moderate to high potential to detect change. We also confirmed high sensitivity to change for DAS28 in this study, in line with our previous observations. [3] The relatively high values for DAS28 in the two anti-TNF cohorts compared to the rituximab cohort, might perhaps be explained in part by the early effects of TNF inhibitors on the central nervous system, [16] affecting the patient reported outcomes. Changes in MRP8/14 levels and DAS28 do suggest an effect of rituximab 4 weeks after the first infusion, but the values are relatively low due to the time point that is early for this specific mechanism of action and due to the fact that no methylprednisolone pre-treatment was given.

The sensitivity to change for serum levels of MRP8/14 resembles that for expression of CD68 positive macrophages in synovial tissue samples in patients treated with effective treatments across different mechanisms of action. [2], [3] Of importance, this soluble biomarker does not appear to be susceptible to placebo effects, expectation bias or regression to the mean in proof of mechanism studies of relatively short duration in contrast to DAS28, with an SRM of 0.58 (moderate sensitivity to change) in the placebo/ineffective treatment studies. The SRM of MRP8/14 was low in EULAR responders after ineffective/placebo treatment, which supports the notion that this biomarker is not susceptible to placebo effects in studies of short duration.

The test characteristics of CRP were also clearly not as good as those for MRP8/14 in discriminating between effective and ineffective treatment. To exclude the possibility that this results could be explained by a relatively low CRP level at baseline in some of the patients, we calculated the SRM for CRP excluding all the cases with relatively low levels with a similar result.

Taken together, the results suggest that serial measurement of MRP8/14 levels in peripheral blood could be used as a biomarker to screen for initial evidence of efficacy on the group level during early drug development. This study provides the rationale for future validation studies using known effective drugs with a distinct mechanism of action such as tocilizumab, abatacept and tofacitinib compared to placebo and ineffective experimental treatments.
